# Transdiagnostic associations between subjective gesture behaviour and objective performance in schizophrenia and depression

**DOI:** 10.1038/s41398-026-04059-6

**Published:** 2026-04-24

**Authors:** Anastasia Pavlidou, Sofie von Känel, Lydia Maderthaner, Alexios Malifatouratzis, Victoria Chapellier, Petra V. Viher, Hanta Bachofner, Katharina Stegmayer, Grit Hein, Kristina Adorjan, Sebastian Walther

**Affiliations:** 1 University Hospital of Psychiatry and Psychotherapy Bern, Translational Research Centre, University of Bern, Bern, Switzerland; 2 Graduate School for Health Sciences, University of Bern, Bern, Switzerland; 3 Department of Basic and Clinical Neuroscience, Institute of Psychiatry, Psychology and Neuroscience, King’s College London, London, United Kingdom; 4 Competence Centre for Psychosomatics, Department of Neurology, University Hospital Inselspital Bern, Bern, Switzerland; 5 Translational Social Neuroscience Unit, Department of Psychiatry, Psychosomatics, and Psychotherapy, University of Würzburg, Würzburg, Germany; 6 Department of Psychiatry, Psychosomatics, and Psychotherapy, Centre of Mental Health, University Hospital of Würzburg, Würzburg, Germany

**Keywords:** Psychiatric disorders, Diagnostic markers

## Abstract

Social communication deficits are common across mental-health disorders, yet little is known about how individuals perceive their own gesture behaviours. Gaining insight into this, particularly across different disorders, could enhance our understanding of social communicative impairments and disruptions in self-awareness. The current study included 274 participants: N = 113 with schizophrenia, N = 65 with depression and N = 96 healthy controls where we compared self-reported gesture behaviours in social and non-social contexts. These self-reports where further explored in relation to objective-measures of gesture performance and expert-rating scales of symptom severity and social functioning. Both patient groups self-reported impairments compared to controls, but with disorder-specific profiles. Specifically, people with schizophrenia uniquely reported reduced gesture perception and use, while both patient groups reported diminished social gesture production. The schizophrenia group also reported elevated social perception relative to the other groups. The depression group consistently rated themselves higher than the schizophrenia group across domains. Furthermore, only the schizophrenia group showed distinct associations: self-reported social perception was negatively associated with self-reported gesture perception, but positively associated with self-reported social production. Notably, only schizophrenia showed a significant link between self-reported difficulties in social gesture production and objective gesture performance deficits. These findings suggest that disruptions in self-awareness of gesture behaviours manifests differently across disorders and underscore the value of integrating self-report measures together with objective assessments to capture the complexity of social-communicative impairments. This will help in designing tailored interventions aimed at enhancing social communication and awareness in diverse mental-health disorder populations.

## Introduction

As humans, we rely heavily on correctly interpreting nonverbal cues for successful social communication. Gestures are visible bodily movements of the hands, sometimes accompanied by movements of the head and face, used during communication, such as pointing to indicate where something is or to display the size of an object, and they are particularly important as they provide crucial information that can facilitate learning, thinking, and understanding [1, 2]. Accordingly, growing evidence in clinical populations suffering from schizophrenia and depression show that gesture deficits play a crucial role in patients’ overall functioning and quality of life [3, 4]. While schizophrenia and depression share overlapping clinical characteristics [5, 6], researchers have noted distinct differences in gesture impairments in these clinical populations that might be related to each disorder’s unique symptoms and clinical course [7, 8].

Specifically, schizophrenia is a severe, heterogenous mental disorder characterized by a wide variety of symptoms including positive and negative symptoms, motor abnormalities, as well as neurocognitive and socio-cognitive deficits [9], all of which are associated with impairments in gesture perception and production [10–13]. In addition, both first and multiple-episode people with schizophrenia and those at high-risk for psychosis are known to use fewer or incorrect gestures during social interactions and spend significantly less time observing other peoples’ gestures [14–17]. Poor hand gesture performance is a predictor of poor functional outcome and is often associated with deficits in other nonverbal cues, such as gestural knowledge, tool use, emotional prosody, and facial expressions, suggesting a generalized impairment in nonverbal communication rather than an isolated one in schizophrenia [4, 8]. This indicates that gesture impairments in this population align with other socio-cognitive domains, while at the same time placing greater demands on action understanding and the integration of kinematic and semantic information [18, 19]. These gesture deficits are also linked to structural and functional aberrations in motor and language brain areas important for correct gesture processing [20–27].

In contrast, depression is primarily a mood disorder, characterized by persistent feelings of emptiness, sadness and irritability coupled with bodily and cognitive alterations that significantly affect patients’ functioning and well-being [28, 29]. While gesture research in depression is not as extensive as in schizophrenia, it nonetheless suggests impairments and less spontaneous gesture use [3, 30, 31]. However, the observed impairments often appear to be specific to particular tasks, and associated with specific processes [7, 32]. For example, a study examining gesture performance in depression showed that patients performed poorer than healthy controls in categories where correct gesturing is strongly dependent on working memory abilities [7]. When working memory processes were not necessary for successful execution, performance was similar to that of healthy controls [7]. This observation aligns with other social domains such as mental state inference and facial affect, in which performance becomes worse not only with greater depression severity but also with higher cognitive demands [33, 34]. Further, poor gesture performance in depression seems to be independent from symptom severity, motor abnormalities or gestural knowledge, unlike schizophrenia [7, 8, 35, 36]. Likewise, studies examining brain activity during gesture interpretation and recognition of meaningful gestures in people with depression did not imply aberrancy, but rather to the existence of compensatory mechanisms reflected by the recruitment of brain areas important for neurocognition to successfully complete the required tasks [37–40].

Although gesture impairments have been well documented in schizophrenia and depression through standardized, video supported, objective assessments, self-report measures of gesture behaviour remain scarce, despite their emerging prognostic value [41]. Self-report measures evaluate one’s self-awareness into their own feelings, experiences and behaviors, an inherently complicated concept that relies on the multisensory integration of multiple factors [42]. When one or more of these factors is manipulated the perception of the self and its relation to the outside world changes [43–45].

Schizophrenia and depression are both characterized by disturbances in self-experience and social interaction [46–48], yet little is known about how individuals with these disorders perceive their own nonverbal communication behaviours. Importantly, both disorders are associated with negative processing biases that may affect how they perceive and interpret social cues, which may be reflected in self-reports of gesture behaviour by magnifying perceived communicative difficulties or misinterpreting ambiguous interactions as more negative [49–53]. Understanding self-evaluation in this context is clinically meaningful: if individuals are unaware of their communicative difficulties, it may hinder treatment engagement, social integration, or rehabilitation outcomes. While self-report is often questioned for its reliability in clinical populations, especially where insight may be impaired, it remains a valuable tool for capturing subjective experience, particularly when interpreted alongside performance-based measures.

Our group has previously demonstrated links between gesture performance and perception in schizophrenia [8], providing evidence that both producing and interpreting gestures are affected. However, the current study moves beyond external performance to focus on self-evaluation of gesture abilities using the Brief Assessment of Gestures (BAG) questionnaire [54]. Specifically, the BAG examines four dimensions of gesture abilities including how often people notice others’ gestures (gesture perception), use gestures while speaking (gesture production), emotionally respond to others’ gestures (social perception) and rely on them in demanding social situations like language barriers (social production). Unlike earlier work, this approach allows us to directly assess how patients perceive their own gestural and social-communicative behaviour, which is a step towards understanding the internal experience of social dysfunction in mental illness, that cannot be captured from clinical observation alone.

To our knowledge, only one prior study has explored self-assessed gesture skills across diagnostic groups [41]. However, the study included individuals at clinical high-risk for psychoses and internalized disorders and was not specific to schizophrenia and depression and did not account for the social dimensions of gestures [41]. To this end, the current study directly compares self-reported gesture processing across schizophrenia, depression, and healthy controls using the BAG. We also examined how these self-reports relate to objective gesture performance, symptom severity, and social functioning. We hypothesize that both patient groups will show reduced self-assessment of gesture skills, with distinct patterns emerging between schizophrenia and depression, particularly in domains involving social aspects of gestures.

## Materials and methods

### Participants

The current study included a total of 274 participants. Data were combined from four different studies (two completed [7, 55] and two ongoing (BASEC 2021-02047 and 2023-00309) and included 113 people with schizophrenia, 65 people with depression, and 96 healthy controls. Most participants (269/274; 98%) were right-handed in accordance to the Edinburgh Handedness Inventory [56]. All participants spoke fluent German, lived in Switzerland and understood the nature of the tasks. Patients were recruited from the inpatient and outpatient departments of the University Hospital of Psychiatry and Psychotherapy in Bern, Switzerland, and diagnosed with schizophrenia spectrum disorders (98/113; 87% schizophrenia and 15/113; 13% schizoaffective disorder) or major depressive disorder in accordance with the Mini International Neuropsychiatric Interview and DSM-5 criteria. At the time of testing, 87% (98/113) of our participants with schizophrenia were on antipsychotic medication and 92% (60/65) of our participants with depression were on antidepressant medication. Healthy controls were recruited from advertisements and word-of-mouth. Inclusion criteria for all participants were: 18–65 years of age, no substance abuse with the exception of nicotine, and no history or current neurological disorders associated with movement impairments. Additionally for healthy controls, exclusion criteria included personal and first-degree relatives’ diagnosis of any psychiatric disorders. All participants provided written informed consent and all studies were approved by the Kantonale Ethikkommision (KEK) Bern and thus comply with the tenets of the declaration of Helsinki. Demographic and clinical characteristics for all three groups can be found in Table 1.

**Table 1:** Demographic and clinical characteristics.

	Schizophrenia Patients (n = 113)	Depression Patients (n = 65)	Healthy Controls (n = 96)	Statistic; p-value
Age (years)	38.3 ± 1.1	35.8 ± 1.4	34.9 ± 1.3	5.5; 0.06
Sex (% females)	43.3	56.9	54.2	3.9; 0.1
Education^a^	13.6 ± 0.6	15.1 ± 0.4	15.9 ± 0.3	31.1; <0.0001*
				Schizophrenia vs Controls: 5.5; < 0.0001*
				Depression vs Controls: 1.9; 0.05
				Schizophrenia vs Depression: 2.9; 0.005*
PANSS Total	67.0 ± 1.9	/	/	/
MADRS Total	/	26.8 ± 1.1	/	/
Mean BAG total	3.1 ± 0.1	3.2 ± 0.1	3.4 ± 0.1	14.5; 0.0006*
				Schizophrenia vs Controls: 3.8; 0.0005*
				Depression vs Controls: 2.3; 0.03*
				Schizophrenia vs Depression: 0.9; 0.3
TULIA Total^b^	191.4 ± 2.1	205.8 ± 1.4	215.7 ± 0.9	95.2; < 0.0001*
				Schizophrenia vs Controls: 9.8; < 0.0001*
				Depression vs Controls: 4.5; < 0.0001*
				Schizophrenia vs Depression: 4.6; < 0.0001*
SOFAS score^c^	49.7 ± 1.3	52.1 ± 1.8	97.4 ± 0.3	148.6; < 0.0001*
				Schizophrenia vs Controls: 11.6; < 0.0001*
				Depression vs Controls: 9.5; < 0.0001*
				Schizophrenia vs Depression: 0.8; 0.4

Values represent the mean ± SEM for each group.

BAG Brief Assessment of Gestures, MADRS Montgomery-Åsberg Depression Rating Scale, PANSS Positive and Negative Syndrome Scale, SOFAS Social and Occupational Assessment Scale, TULIA Test of Upper Limb Apraxia.

^a^one patient with depression missing education.

^b^26 patients with schizophrenia and 12 controls are missing TULIA.

^c^28 controls missing SOFAS.

*denotes a significant difference between groups.

### Clinical and functioning assessments

To assess symptom severity at the time of testing we used the *Positive and Negative Syndrome Scale* (PANSS [57]) for our participants with schizophrenia and the *Montgomery-Åsberg Depression Rating Scale* (MADRS [58]) for our participants with depression. In addition, we used the *Social and Occupational Functioning Assessment Scale* (SOFAS [59]) to evaluate all our participants’ individual level of social and occupational functioning. 28/96 (29.2%) controls were missing the SOFAS assessment.

### Gesture perception and production

#### Subjective evaluation

We used the *Brief Assessment of Gestures* (BAG [54]) a self-rating scale which includes 12-statements of everyday communicative situations to understand how participants evaluate their own experiences related to gesture perception and gesture production processes along with their social components. Participants are asked to evaluate each statement using a 1 (not agree) – 5 (fully agree) point Likert scale. Factor analyses revealed that BAG is separated into four distinct subdomains: gesture perception, which includes items referring to the perception of gesture information such as ‘*I find it very annoying when I’m talking to someone who gestures a lot when they talk*’; gesture production which involves items referring to the amount of gestures produced such as ‘*I’ve been told before that I gesture a lot when I talk*’; social perception which refers to items involved with the empathetic properties of gesture perception, like emotional valence and perspective-taking characteristics such as ‘*When I see someone gesturing a lot, I often wonder if I would have used the same gestures*’; and finally, social production which refers exclusively to social situations where participants have to infer the mental states of others and use gestures directly or as a supporting tool to facilitate social communication such as ‘*When talking in noisy places, I often gesture a lot to make myself understood over the noise*’. Values of 4/12 of the statements were recoded to account for the negative wording (i.e. *‘I find it very annoying…’*). Higher scores on the BAG are indicative of superior gesture use.

#### Objective gesture performance

We used the *Test of Upper Limb Apraxia* (TULIA [60]) to objectively measure gesture production abilities respectively for all participants. In short, the TULIA includes 48 items and investigates participants’ ability to accurately perform gestures following visual demonstration from the experimenter (imitation domain) and following verbal command from the experimenter (pantomime domain). Each domain includes three categories of gestures: meaningless (novel gestures without any semantic properties), intransitive (highly communicative gestures) and transitive (tool-based gestures). The TULIA test involves the experimenter and participant sitting vis-à-vis with both placing their hands flat on the table between them. The test is video-recorded and later quantified according to the scoring manual by independent examiners who is blind to the groups. For this study, TULIA scoring was performed by two trained raters following standardized procedures. The primary rater who is the principal investigator (S.W.), received formal training directly from the test developers and independently quantified 28 of the 236 total TULIA assessments. The principal investigator subsequently provided direct training and ongoing supervision to the second rater, a postgraduate neuroscientist (A.P.) prior to the start of scoring. The second rater completed the remaining assessments under this standardized structure. The TULIA score ranges from 0 to 240 with higher scores indicative of superior gesture performance. TULIA data were missing for 26/113 people with schizophrenia (23.0%) and for 12/96 controls (12.5%).

#### Data analyses

The mean rating scores for each group and each BAG subdomain were calculated with scripts written in RStudio (version 2025.09.0 + 387). We used linear mixed models adjusted for education to evaluate self-reported differences between groups for each BAG subdomains using the lmer function from the *lme4* package (version 1.1-36). Group (schizophrenia, depression, and healthy controls) and BAG subdomains (gesture perception, gesture production, social perception and social production), as well as, their interactions were added as fixed effects. Since education was significantly different between our groups we added is a covariate in our model. In addition, a single intercept parameter calculated for each participant was added as random effects to account for repeated measures. We assessed potential collinearity among the BAG subdomains by computing their variance inflation factor (VIF) using the vif function from the *car* package (version 3.1-3). The adjusted VIF for the BAG subdomains was 4.82 which is below the commonly used threshold of 5 [61], indicating no problematic multicollinearity among the BAG subdomains. Pairwise comparisons were conducted using Holm-Bonferroni adjustments to control for multiple comparisons, using the emmeans function from the package *emmeans* (version 1.10.6). The effect sizes were calculated using the eff_size function from the same package and were defined as standardized marginal mean differences using the model residual standard deviation. The Kruskal-Wallis and chi-square tests were used to assess differences in demographic characteristics (e.g. age and sex), as well as, BAG mean total, TULIA performances and SOFAS ratings between the three groups, and the Dunn’s test using the dunnTest function from the *FSA* package (version 0.10.0) to determine which specific group pairs significantly differed, adjusting for multiple comparisons using the Benjamini-Hochberg method. To explore the relationship between subjective gesture behavior and objective measures of gesture performance, social functioning and symptom severity we applied correlation analyses using the Spearman method again adjusting with Benjamini-Hochberg separately for each group using the cor function from the *stats* package (version 4.4.2). Model-estimated effects were visualized using the *ggplot2* package (version 3.5.1), and correlation matrices were plotted using the *corrplot* package (version 0.95).

## Results

### Demographic and clinical characteristics

Overall, our three groups did not differ in gender but had borderline difference in age and a significant difference in education with controls having more years of education (Table 1). Symptom severity for schizophrenia and depression was mild to moderate and both groups exhibited severe impairments in social functioning as assessed by SOFAS and task-based gesture performance as quantified by TULIA compared to controls. People with schizophrenia performed gestures worse than people with depression (z = 4.6; p-value < 0.0001), while social functioning did not differ between patient groups (z = 0.8; p-value = 0.4; Table 1).

### BAG subdomains between groups

**Fig. 1:**
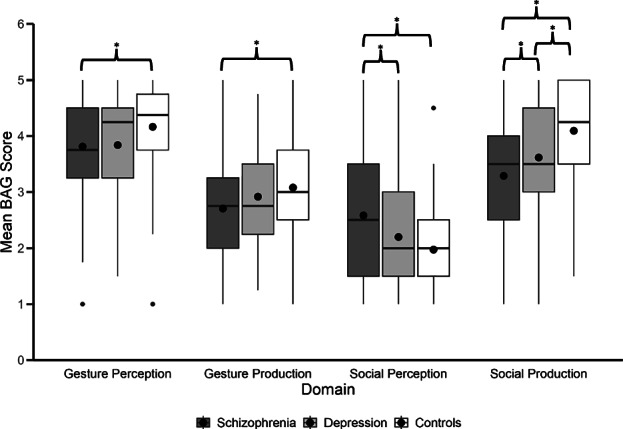
Results. Box plots comparing self-evaluation of the BAG scale across its four domains for schizophrenia (dark grey), depression (light grey) and control (white) groups. The median is represented by the thick horizontal line inside the box and the mean is represented by the black dot inside the box. The upper and lower bound of each box represent the 75th and 25th percentiles of the distribution, while the top and bottom ends of the whisker represent the 95th and 5th percentiles of the distribution, respectively. *denotes a significant difference between groups; the Gesture Perception domain difference reached nominal significance (p = 0.05).

People with schizophrenia reported lower gesture perception and use than people with depression and controls in the BAG (Table 1). Linear mixed models adjusting for education revealed a significant main effect of Group (F = 3.1; p-value = 0.04) and BAG subdomains (F = 199.9; p-value < 0.0001) with Education as a significant covariate in the model (F = 4.1; p-value = 0.04). Importantly, a significant interaction between Group-by-BAG subdomains (F = 12.3; p-value < 0.0001; Fig. 1) was observed. Post-hoc comparisons using the Holm-Bonferroni method revealed that people with schizophrenia reported less gesture perception reflecting inferior perceptual tolerance than controls (ΔEMM = 0.31, 95% CI { − 0.004, 0.62}, *d* = 0.29; Fig. 1) with a weaker but similar pattern observed between controls vs. depression (ΔEMM = 0.28, 95% CI {−0.07, 0.64}, *d* = −0.03; Fig. 1). In contrast, no significant effects were observed between schizophrenia and depression (ΔEMM = 0.02, 95% CI {−0.31, 0.36}, *d* = 0.32). For the gesture production domain schizophrenia again reported significantly less gesture production (ΔEMM = 0.33, 95% CI {−0.01, 0.64}, *d* -= 0.07; Fig. 1) than controls, while no significant effects were observed between controls and depression (ΔEMM = 0.13, 95% CI {−0.22, 0.49}, *d* -= 0.22) or between patient groups (ΔEMM = 0.02, 95% CI {−0.31, 0.36}, *d* = 0.32). Furthermore, people with schizophrenia reported higher social perception suggesting a heightened emotional response to others’ gestures compared to controls (ΔEMM = −0.64, 95% CI {−0.96, 0.33}, *d* = 0.22; Fig. 1) and subjects with depression (ΔEMM = −0.42, 95% CI {−0.76, −0.08}, *d* = −0.26; Fig. 1) while no differences were observed between depression and controls (ΔEMM = −0.22, 95% CI {−0.58, 0.13}, *d* = 0.48). For the social production subdomain, both people with schizophrenia (ΔEMM = 0.76, 95% CI {0.44, 1.07}, *d* = 0.18; Fig. 1) and depression (ΔEMM = 0.46, 95% CI {0.10, 0.82}, *d* = −0.34; Fig. 1) reported less frequent use of gestures in socially demanding situations where verbal communication is hindered compared to controls. Additionally, a significant difference between the patient groups was also observed with the schizophrenia group reporting less gesture use than the depression group (ΔEMM = 0.30, 95% CI {−0.04, 0.64}, *d* = 0.53; Fig. 1).

Primary results were unchanged when study was included as a random intercept to account for pooling across studies (see Supplementary Table S1 for group by study counts). In a further sensitivity analysis additionally adjusting for age and sex alongside education, the BAG subdomains and Group-by-BAG subdomains remained significant while the Group effect was weakened to trend-level significance (see Supplementary Materials).

### Association between BAG self-report scores and objective measures

**Fig. 2:**
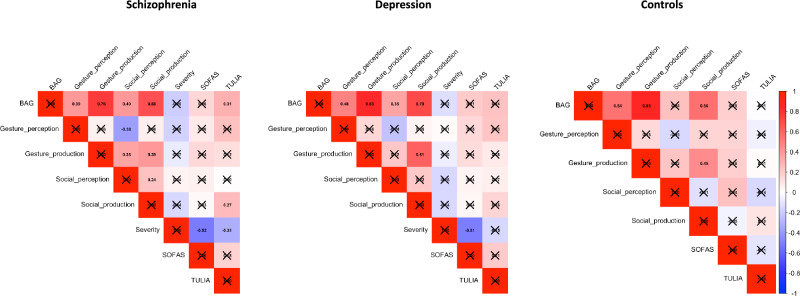
Correlation Matrices between BAG subdomains and objective measures for schizophrenia, depression and controls. Positive correlations are presented in red and negative correlations in blue. The color intensity is proportional to the strength of the spearman correlation coefficient. X represent non-significant correlations. BAG – Brief Assessment of Gestures; Severity (Positive and Negative Syndrome Scale for schizophrenia; Montgomery-Åsberg Depression Rating Scale for depression); SOFAS – Social and Occupational Functioning Assessment scale; TULIA – Test of Upper Limb Apraxia.

Within the domains of the BAG, the group with schizophrenia showed distinct patterns of association. Specifically, the social perception domain was negatively correlated with the gesture perception domain (rho = −0.38; p-value = 0.0008; Fig. 2), but positively correlated with the gesture production domain (rho = 0.36; p-value = 0.0009; Fig. 2). Additionally, the social production domain was positively correlated with both the gesture production (rho = 0.39; p-value = 0.0008; Fig. 2) and social perception (rho = 0.24; p-value = 0.04; Fig. 2) domains. In contrast, for the depression and control groups, the only significant association observed was a positive correlation between the social production and gesture production domains of the BAG (all rho > 0.45; all p-values < 0.0001; Fig. 2).

When examining relationships between subjective and objective measures, the BAG mean total score showed a positive correlation with the TULIA total score in subjects with schizophrenia (rho = 0.31; p-value = 0.01; Fig. 2), which was domain-specific to the social production domain (rho = 0.27; p-value = 0.03; Fig. 2). This suggests that self-reported gesture difficulties mirror objective gesture impairments. These associations were not observed in the depression or control groups (Fig. 2).

In addition, only in the schizophrenia group did symptom severity negatively correlate with TULIA (rho = −0.31; p-value = 0.01; Fig. 2). It is also worth mentioning that symptom severity was strongly negatively correlated with the SOFAS score in both patient groups (all rho > −0.51; all p-values < 0.0001; Fig. 2), which is an expected finding as psychopathology often reduces social and occupational functioning.

## Discussion

The current study investigated how individuals with schizophrenia, depression, and healthy controls perceive their own gesture behaviours in everyday communication, based on their own self-reports. Specifically, we examined four aspects of gesture behaviours: how often people notice gestures used by others (gesture perception); how frequently they use gestures themselves while speaking (gesture production); how much they use gestures in socially demanding situations such as noisy environments or language barriers (social production); and finally, how they emotionally respond to or reflect on others’ gestures (social perception). These self-reports were also compared to objective measures of gesture performance, clinical symptoms, and social functioning. A significant Group-by-BAG subdomain interaction showed that only individuals with schizophrenia reported significantly lower gesture perception and production reflecting lower perceptual tolerance and gesture use compared to healthy controls. However, both patient groups reported using fewer gestures in socially demanding situations (lower social production) than controls, though the depression group scored higher than the schizophrenia group. Interestingly, people with schizophrenia reported higher subjective emotional salience and reflection towards others’ gestures (social perception) compared to depression and healthy controls. In this group, social perception was negatively related to gesture perception (i.e., those who were more emotionally affected by gestures tended to notice them less), but positively related to social production. These patterns were not observed in the depression or control groups. Finally, among individuals with schizophrenia, self-reported gesture difficulties, particularly in the social production domain, were significantly related to objectively measured gesture performance (as assessed by TULIA), and overall gesture self-report scores were associated with symptom severity. This suggests that, in schizophrenia, how patients perceive their own gesture use in communication may reflect meaningful clinical features, including motor expression and symptom severity.

Self-reported deficits in the gesture perception and production domain among individuals with schizophrenia compared to controls likely reflect both subjective social discomfort and altered self-awareness of nonverbal communication in this population. This resonates with existing evidence showing that people with schizophrenia often misinterpret nonverbal social cues and tend to use fewer gestures or in the wrong context during social communication [8, 11, 15, 16, 62, 63]. In previous studies, such deficits have been attributed to dysfunction in the neural mechanisms responsible for gesture perception and production, which include the mirror neuron system and frontal-temporal regions critical for gesture comprehension of semantic and symbolic contexts [20–24, 27, 64, 65]. However, it is important to note that while informative BAG assesses subjective gesture behaviour and thus cannot be interpreted as a direct measure of motor-praxis dysfunction. In contrast, the difference observed between people with depression and controls in the gesture perception domain was more modest and showed weaker statistical evidence suggesting a subtler, less pronounced impact on these abilities. Previous research supports this notion as deficits in the perception of social cues including gestures in this clinical population are often linked to emotional and cognitive biases and altered attentional capacity rather than impairments in the basic perception of gestures [32, 66, 67]. Further, negative processing biases, which are well documented in both disorders, may further influence how patients perceive and evaluate their own gesture behaviours, especially in the presence of ambiguous cues [50, 53].

In evaluating the social production and perception domains of BAG we observed distinct patterns both between patient and control groups and among our patient groups suggesting that disorder-specific characteristics may contribute to the observed differences in domains that are more socially relevant. Specifically, individuals with schizophrenia report being more subjectively affected by or emotionally engaged with other peoples’ gestures as reflected in higher ratings on the social perception domain. Meanwhile, they report using such gestures less themselves, especially in socially demanding situations as seen in the social production domain consistent with previous observational studies showing reduced nonverbal expressiveness in individuals with schizophrenia during social interaction [63]. This juxtaposition reflects a dissociation between social perceptual salience and social expressiveness and empathy in schizophrenia. While patients might experience heightened subjective emotional salience to others’ gestures, which is most likely driven by aberrant salience attribution, a common feature in schizophrenia, production of gestures requires intact motor planning and execution, motivational drive, expressivity, and theory of mind. All of these processes are significantly impaired in subjects with schizophrenia [36, 68–74]. Such aberrant salience attribution may also reflect a broader negative processing bias whereby socially relevant cues are experienced more intensely, contributing to the heightened ratings in the social perception domain without necessarily reflecting accurate interpretations of others’ gestures [69, 75], and potentially hindering effective communicative gesture use. In contrast, people with depression reported social perception ratings comparable to controls, suggesting that they do not exhibit aberrant salience towards others’ gestures. However, despite this preserved perceptual capacity, ratings of social production were reduced compared to controls. Recent evidence suggests that neurocognitive impairments are prevalent and highly associated with aberrant gesture production in depression [7, 76]. Producing gestures, especially in highly demanding social situations requires real-time coordination of cognitive resources such as planning motor sequences and responding to social cues [77–79] making gesture production more arduous and less frequent in depression but not as profoundly impaired as in schizophrenia. Taken together, schizophrenia reflects a breakdown in social-perceptual integration while depression might reflect reduction in gesture use due to cognitive effort.

The mismatch between heightened subjective emotional salience of others’ gestures and reduced use of gestures in socially demanding situations is further supported by a unique pattern observed only in the group with schizophrenia: a significant association between social perception and social production domains. While these two aspects were unrelated in the other groups, individuals with schizophrenia who reported greater subjective emotional salience to others’ gestures also tended to report more gesture use in socially demanding contexts. This finding suggests that for some individuals with schizophrenia, heightened social perception may drive compensatory attempts at expression, even if overall gesture use remains impaired, or may reflect negative processing biases that interfere with accurate social interpretation [53, 80]. Disruptions in self-other processing and altered connectivity within the brain’s social cognition network may underlie this atypical relationship, potentially contributing to both the heightened subjective emotional salience of others’ gestures and the struggle to translate that awareness into effective nonverbal communication [80–82]. This disrupted integration is further substantiated by the negative correlation between gesture and social perception domains also observed only in schizophrenia, suggesting that greater sensitivity to others’ gestures, potentially driven by aberrant salience appears to interfere with rather than support social understanding and awareness [83–85]. In contrast, the association between gesture production (how much people gesture in general) and social production (use of gestures in socially demanding scenarios) observed in all groups suggests a fundamental and robust relationship that is less sensitive to psychopathology.

Exclusive to schizophrenia, was also the correlation between the social production domain and TULIA, a well-established standardized test that evaluates gesture production of meaningful and meaningless gestures. This association suggests that self-reported difficulties in gesture use in schizophrenia during highly demanding social scenarios may be partially related to objectively measurable impairments in motor planning and execution especially when incorporating gestures into communicative actions, reflecting disruptions in the praxis network which is responsible in supporting gesture planning and execution [11]. However, due to the subjective nature of BAG, these difficulties should be considered informative rather than indicative of motor-praxis dysfunction as some patients may report substantial difficulties without corresponding TULIA impairments, and vice versa. Further, the association with overall symptom severity and TULIA suggests that these gesture deficits are embedded within the broader clinical and social-cognitive dysfunction linked to schizophrenia in line with previous studies [8, 11, 86, 87]. Notably, despite social production impairments, patients with depression do not show the same associations; perceptual and production domains remain relatively independent likely reflecting cognitive effort rather than socio-cognitive decline.

There are a few limitations that should be discussed. First, while the current study is based on subjective self-reports which may be influenced by negative processing biases affecting self-evaluation and social behaviour, the observed association between the social production domain with our objective gesture performance task (TULIA) supports the validity of these self-reported observations. Second, the cross-sectional and correlational nature of the current study prohibits causal interpretation or determination of directionality of relationships among subjective and objective measures. Longitudinal studies offer significant advantages in how subjective self-awareness in gesture abilities and its relationship to objective measures changes over time. Third, cognitive domains such as processing speed, attention, and executive function were not specifically evaluated. Incorporating broader neurocognitive assessments would provide a more comprehensive understanding of how cognitive dysfunction influences subjective gesture perception and use. Finally, the generalizability of these findings might be limited by our sample characteristics. The severity of our sample’s symptoms, medication effects and illness duration can significantly affect both subjective and objective measures.

In conclusion, our findings highlight the value of integrating self-report and objective task-based measures to better understand gesture-related social functioning in psychiatric disorders. Self-reported experiences of gesture use revealed disorder-specific patterns that may not be captured through observational methods alone. In schizophrenia, a dissociation between heightened subjective emotional salience to others’ gestures and reduced gesture use in socially demanding contexts suggests a breakdown in the integration of social perception and expression. In depression, reduced gesture use likely reflects cognitive effort rather than altered social perception. Specifically, recognizing how distinct disruptions in self-awareness, sense of self, and social identity in schizophrenia and depression uniquely shape subjective experiences and perceptions of social communication can facilitate targeted therapeutic interventions aimed at enhancing social functioning [55, 88] and insight across different psychiatric populations such as social skills training [89], non-invasive brain stimulation [90] and virtual reality [91, 92]. Finally, our results suggest that clinicians should inquire about patients’ perceptions of their own gesture behaviours and understanding to increase knowledge about social-cognitive skills.

## Supplementary information

Supplementary Material

## Data Availability

Participants did not consent to the sharing of their health-related data.
