# Supplementary material for: Transdiagnostic associations between subjective gesture behaviour and objective performance in schizophrenia and depression
Source: Transl Psychiatry. 2026 Apr 24;16:305. doi: 10.1038/s41398-026-04059-6 (PMC13243492; doi:10.1038/s41398-026-04059-6)
Supplement: Supplementary file 1 — Supplementary Material [file 41398_2026_4059_MOESM1_ESM.docx]

**Transdiagnostic Associations Between Subjective Gesture Behaviour and Objective Performance in Schizophrenia and Depression**

Anastasia Pavlidou, Sofie von Känel, Lydia Maderthaner, Alexios Malifatouratzis, Victoria Chapellier, Petra V. Viher, Hanta Bachofner, Katharina Stegmayer, Grit Hein, Kristina Adorjan, Sebastian Walther

**Supplementary materials**

**Results**

A sensitivity analysis was conducted using a linear mixed-effects model with **Group**, BAG sub**domain**, and their interaction as fixed effects, adjusting for **Age**, **Sex**, and **Education**. The **main effect of Group** was weakened to **trend-level significance** (F = 2.4, p = .09). In contrast, **BAG subdomain** remained strongly significant (F = 199.9, p < .0001), and the **Group-by-BAG subdomain interaction** remained statistically significant (F = 12.3, p < .0001), indicating that group differences depended on domain. Among covariates, **Age** (F = 9.41, p = .002), and **Education** were significant (F = 4.4, p = .03), while **Sex** was at trend-level (F = 3.1, p = .08). Post hoc comparisons using the Holm-Bonferroni method revealed no pairwise group differences in the gesture perception domain that reached statistical significance. Both schizophrenia (ΔEMM = .27, 95% CI {−.04, .58}, d = .34) and depression (ΔEMM = .28, 95% CI {−.07, .64}, d = .01) did not differ from controls when reporting their perceptual tolerance of gestures. Similarly, both patient groups reported similar perceptual tolerance to each other (ΔEMM = −.01, 95% CI {−.35, .33}, d = .32). For the gesture production domain, the schizophrenia group reported using less gestures than controls, although the contrast was at trend level (ΔEMM = .29, 95% CI {−.02, .60}, d = -.04), while the depression group reported no difference in the frequency of gestures use from controls (ΔEMM = .13, 95% CI {−.23, .49}, d = -.19), and the two patient groups did not differ from one another (ΔEMM = .16, 95% CI {−.18, .50}, d = .14). For the social perception domain, the schizophrenia group reported higher emotional response from both controls (ΔEMM = −.69, 95% CI {−1.00, −.38}, d = .27) and depression (ΔEMM = −.46, 95% CI {−.80, −.12}, d = -.26), while the depression reported similar emotional response to controls (ΔEMM = −.23, 95% CI {−.59, .13}, d = .53). Finally, for the social production domain, both schizophrenia (ΔEMM = .72, 95% CI {.41, 1.04}, d = .23) and depression groups reported less gesture during socially demanding situations (ΔEMM = .46, 95% CI {.11, .82}, d = -.30), while the difference between the two patient groups was weakened to trend-level differences with the depression group reporting higher gesture use than schizophrenia (ΔEMM = .26, 95% CI {−.08, .60}, d = .53).

Table S1: Sample sizes by group and study

|  | Controls | Depression | Schizophrenia |
| --- | --- | --- | --- |
| Study 1 | 28 | 35 | 0 |
| Study 2 | 25 | 30 | 0 |
| Study 3 | 31 | 0 | 87 |
| Study 4 | 12 | 0 | 26 |
